# HLA Typing and Celiac Disease in Moroccans

**DOI:** 10.3390/medsci5010002

**Published:** 2017-01-06

**Authors:** Daniela Piancatelli, Imane Ben El Barhdadi, Khadija Oumhani, Pierluigi Sebastiani, Alessia Colanardi, Abdellah Essaid

**Affiliations:** 1 National Research Council (CNR)-Institute of Translational Pharmacology, U.O.S. L’Aquila, Via Carducci 32, 67100 L’Aquila, Italy; pierluigi.sebastiani@cnr.it (P.S.); alessia.colanardi@cnr.it (A.C.); 2 Mohammed V-Souissi University, 10000 Rabat, Morocco; ouhabiimane@yahoo.fr (I.B.E.B.); feydi2001@yahoo.fr (A.E.); 3 Medicine C, Department of Gastroenterology, Ibn Sina Hospital, 10000 Rabat, Morocco; 4 Institute National d’Hygiene, 10000 Rabat, Morocco; khaoumhani@gmail.com

**Keywords:** celiac disease, HLA, Morocco, population, North Africa

## Abstract

Genetic and environmental factors are responsible for differences in the prevalence of some diseases across countries. Human leukocyte antigen (HLA) allele frequencies in North African populations show some differences in their distribution compared to Europeans, Mediterraneans, and sub-Saharans, and some specific alleles and haplotypes could be clinically relevant. Celiac disease (CD) has been fast increasing in prevalence in North Africa; but few immunogenetic data are available for this area, in which a high prevalence of the disease has been described. In this report, we assess and discuss results of HLA class II (HLA-DQA1/DQB1/DRB1) typing in Moroccan patients with CD and compare them with a control population from Morocco—genetically well characterized—and with other North African, Mediterranean, and European populations. The classical HLA-DQ associations were confirmed in Moroccans with CD. The high frequency of DQ2.5 homozygosity (45.2%) found in Moroccans with CD was noteworthy as compared with other populations (23%–32%). The genetic risk gradient for CD, identified by previous studies, has been confirmed in Moroccans with some differences, mainly concerning DQ8 genotypes. This study provides the immunogenetic framework of CD in Moroccans and confirms the need to learn more about associations with additional HLA and non-HLA genetic factors.

## 1. Introduction

Celiac disease (CD) is a multifactorial and autoimmune disease caused by a dysregulated immune response to wheat gliadin, which develops in genetically predisposed individuals. CD shows a prevalence of 0.5%–1% in Caucasian populations from Europe and North America; prevalence is increasing in developing countries, especially in North Africa and the Middle East, and at present it is a common disorder in North Africa [1]. It was hypothesized that genetic factors (i.e., the very high frequency of human leukocyte antigen (HLA) predisposing haplotypes, DQ2 and DQ8) joint with a fast increase of gluten intake are the main reasons for this condition, although recently further hypotheses are emerging to explain differences in geographical distribution of the disease. HLA genes encode highly polymorphic transmembrane glycoproteins which play an important role in recognition of foreign antigens; they are key elements in diseases and transplantation. HLA allele distribution is different among populations and HLA polymorphism could be a good marker to study disease susceptibility in HLA-associated autoimmune diseases.

HLA allele/haplotype frequency distribution in North Africans showed some differences from European and Mediterranean populations, while more important differences were found compared with sub-Saharans. The mixing of populations and the high polymorphism of HLA alleles are characteristic of Maghreb [2,3,4,5]. During previous studies, we found that HLA frequencies in this region are intermediate between Sub-Sahara and Europe; some specific HLA alleles and haplotypes that characterize this region could be of interest for clinical purposes.

In CD, HLA class II genetic associations are well defined and the disease rarely develops in absence of specific HLA class II alleles. In the Maghreb area, CD incidence could be associated both with dietary habits and with the high frequency of DR3-DQ2 haplotypes [6]. Among North Africans, immunogenetics of CD has not been deeply studied in Moroccans. In order to evaluate the genetic disease risk in North Africa, CD-associated HLA-DQA1, -DQB1, -DRB1 genotypes and haplotypes were assessed in Moroccan patients with CD and compared with data from North African and Caucasian Mediterranean and European populations.

## 2. Materials and Methods

### 2.1. Population Sample

One-hundred and fifteen DNA samples from Moroccan individuals with CD, 17% men and 83% women, aged 18–67 years, were collected at the Medicine C Service, Ibn Sina Hospital of Rabat. Diagnosis was confirmed by serology (anti-transglutaminase, anti-endomysium antibodies) and intestinal biopsy; HLA class II polymorphisms associated with CD were investigated in the present study. Clinical and anamnestic data of patients were registered and patients with uncertain diagnosis were excluded from the study.

**Figure 1:**
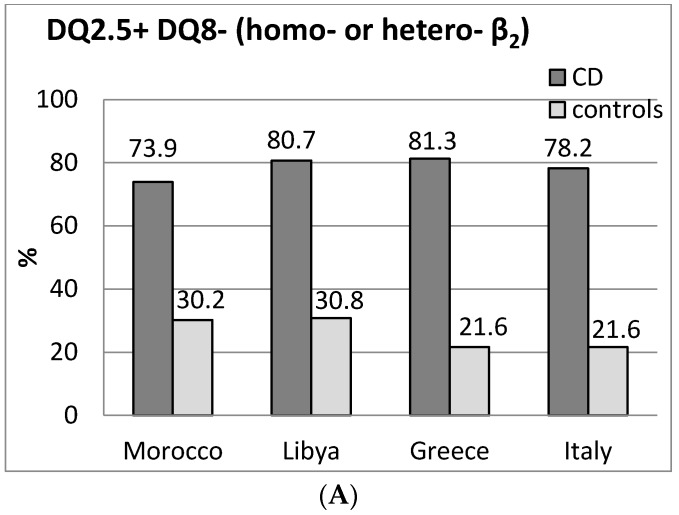

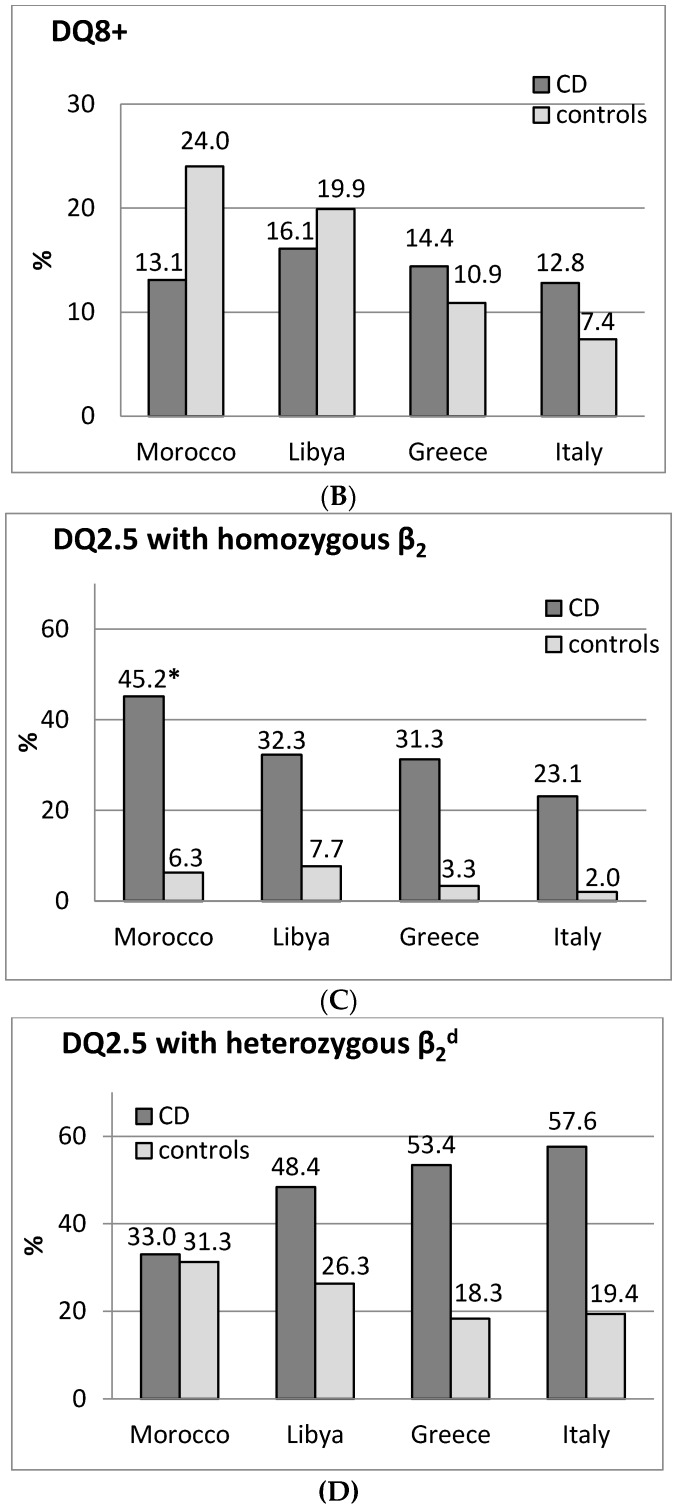
Distribution of HLA-DR-DQ genotypes in celiac disease (CD) and controls from Morocco ^a^, from North Africa ^b^ and from other Mediterranean populations ^c^. Genotype frequencies (%) are reported on the top of the columns. (**A**) frequencies of DQ2.5 positive/DQ8 negative individuals; (**B**) frequencies of DQ8 positive individuals; (**C**) frequencies of DQ2.5 with homozygous β_2_ chain individuals (DQ2.5 homozygous); (**D**) frequencies of DQ2.5 with heterozygous β_2_ chain individuals. * *p* < 0.001 vs. controls. ^d^ Figure 1D includes DQ8+ individuals (DQ2.5/DQ8 genotypes). Population references: ^a^ Present study; ^b^ Lybia [11]; ^c^ Greece [12]; Italy [9].

**Figure 2:**
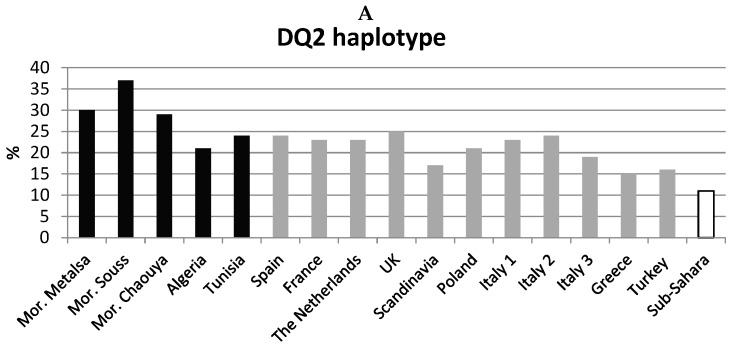

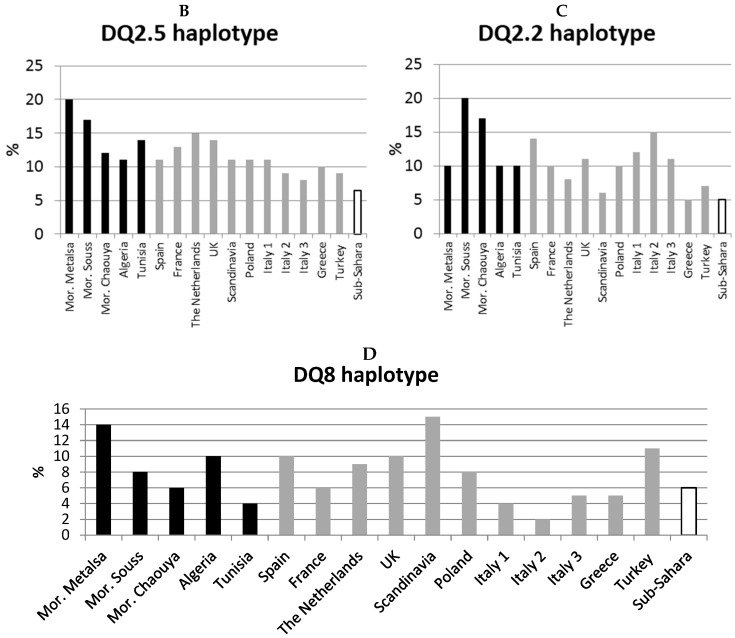
DQ2 and DQ8 haplotype distribution in African and European populations. (**A**) DQ2 haplotype frequencies; (**B**) DQ2.5 haplotype frequencies; (**C**) DQ2.2 haplotype frequencies; (**D**) DQ8 haplotype frequencies. In black: Maghreb populations. In grey: Mediterranean and European populations. In white: Sub-Saharans (average frequency of three populations). References: Mor. = Morocco (Metalsa) [3]; France, Scandinavia, Italy 2 [8]; Greece [12]; Morocco (Souss), Morocco (Chaouya), Algeria, Tunisia, Turkey, Sub-Sahara (Cameroon, Congo, Gabon) [15]; Italy 1 [16]; Spain, The Netherlands, UK, Poland, Italy 3 [17].

Results were compared with a healthy control population from Morocco (Metalsa, North Morocco), previously genetically well characterized by our research group [2,3] and representative of the genetic substratum of Maghreb and with other Mediterranean and European populations (data extracted from previously published articles and from public databases, see Figure 1 and Figure 2 and Tables 1 and 3 for references).

A written informed consent was signed by each patient and the study was approved by the committee of the National Center for Scientific and Technical Research of Morocco, with code 103212 on 2 April 2012, with respect to the ethical principles of the Ministry of Health of Morocco.

### 2.2. DNA Extraction and CD-Associated HLA Class II Polymorphism

Genomic DNA was extracted from peripheral blood using commercial kits (QIAamp DNA blood mini kit, Qiagen, Hilden, Germany). HLA class II alleles associated with celiac disease were identified using a polymerase chain reaction-sequence-specific primer (PCR-SSP) method (DQ-CD Typing kit, Biodiagene s.r.l., Palermo, Italy) [7]. The detected alleles were HLA-DQA1*02:01, *03, *05, HLA-DQB1*02, *03:01/04, 03:02, HLA-DRB1*03, *04, *07, *11, *12, and the DQB1*02 homozygous status.

Patients were stratified according to risk group classifications for CD based on genotypes of HLA-DR and -DQ loci [8,9].

Homozygous DQB1*02 status, detected by the DQ-CD method, included: (1) individuals with the DQ 2.5 haplotype, belonging to the risk group 1 [8] (DR3-DQ2, homozygous DR3-DQA1*05-DQB1*0201); (2) individuals, included in the risk group 1, carrying the DQ 2.5 and DQ 2.2 haplotype (DR3/DR7-DQ2, DR3-DQA1*05-DQB1*0201/ DR7-DQA1*02-DQB1*0202); and (3) individuals with the DQ 2.2 haplotype, included in risk group 4 (DR7-DQ2, homozygous DR7-DQA1*02-DQB1*0202).

DRB1, DQA1, and DQB1 allele data of the control population derived from previous studies on HLA polymorphism in Moroccans. Allele frequencies were in Hardy–Weinberg equilibrium, as previously reported [3] (DQA1, *p* = 1, DQB1, *p* = 0.41; DRB1, *p* = 0.40). CD-associated haplotypes and genotypes in controls were analyzed in the present study.

### 2.3. Statistical Analysis

Allele and genotype frequencies were calculated by direct counting. Differences between groups were analyzed using χ^2^ test; Bonferroni correction was applied (*p_c_*) for multiple comparisons. The odds ratio (OR) and 95% confidence interval (CI) were computed. *p* level was set at 0.05. Disease risk was calculated considering a disease prevalence of 1:100 in the control population, according to reported methods (for each HLA-DQ genotype, percentage of controls divided by percentage of patients multiplied by 100) [9]. Haplotype frequencies were estimated using an expectation–maximization (EM) algorithm for multilocus genotypic data when the gametic phase is not known. For statistical analysis, the SPSS (SPSS Inc., Chicago, IL, USA) and Arlequin [10] software were employed.

## 3. Results

### 3.1. DQ2 and DQ8 genotypes in CD 

Results of Moroccans and population comparisons are shown in Figure 1 and Table 1. In general, the classical HLA class II high risk genotypes found in other populations were confirmed in Moroccan patients with CD (Figure 1A).

In total, 87% of patients with CD carried the DQ2 and/or DQ8 heterodimers (DQA1*05-DQB1*02, DQ2.5, in *cis* or *trans* configuration, and/or DQA1*03-DQB1*0302) vs. 54.2% of controls (*p* < 0.001).

In other populations with CD DQ2, DQ2/DQ8 were 96.8% in Libyans [11], 91% in Italians [9], and 95.8% in Greeks [12].

DQB1*02 allele, in its homozygous configuration (Figure 1C), was seven times more frequent in CD than in the Moroccan controls, *p* < 0.001, confirming previous data [13]; no difference between patients and controls was found as for heterozygous configuration (Figure 1D) and DQB1*0302 allele frequencies (6.5% vs. 12% of the Moroccan controls).

DQ8 frequencies are reported in Figure 1B. As in other populations, DQ8 occurred more frequently in DQ2 negative CD patients (27.8%) than in DQ2 positive (6.3%, *p* = 0.005), while no difference was present in the control group (26.2% vs. 20.0%, *p* = not significant (ns)).

HLA-DQ2.2 heterodimer was present in 45.2% of patients and 19.8% controls (*p* < 0.0001; in 43/115 patients and in six controls it was in association with DQ2.5 or DQ8 heterodimers) (other populations with CD: variable frequencies, 3.2%–20.4%); six patients had only the α5 chain and nine patients only the β2 chain. No Moroccan patients were DQ2/DQ8/β2/α5 negative.

### 3.2. Risk Groups and Risk Gradient for CD

HLA class II genotypes, haplotypes, and corresponding disease risk according to Margaritte-Jeannin [8] and Megiorni [9] classifications are reported in detail in Table 1. All patients with CD had at least one HLA risk allele. Table 1 evidences the risk gradient found in Moroccans according to genotypes. The risk gradient identified by previous studies has been confirmed in Moroccans, with some differences, mainly regarding risk associated with DQ8 genotypes. 

The highest risk for CD, 1:14, was found in risk group 1 (two DQ2, DR3-DQ2 or DR3/DR7-DQ2, with homozygous β2). For HLA-DQ8, a different trend, as compared with Italian [9] and Spanish [14] populations, was evidenced: this is probably because of a different frequency of DQ8 found in Moroccan controls, as in some other populations: in both Moroccans and Libyans, DQ8 genotypes and haplotypes showed a high frequency in the controls, and consequently the CD risk was lower than in other populations for this genotype. This changes the risk for CD of patients with the DQ8 genotype, which seems higher in Italians and in other populations than in Moroccans.

A possible high risk was found in Group 2, DQA1*02:01-DQB1*02-DRB1*07/DQA1*05-DQB1*03:01,04-DRB1*11 (1:10) (α5β2 *trans* configuration) and in DQ2.2 with 2 DQB1*02 (1:59), although, due to the small number of subjects in these two groups, this needs further insights. So, in addition to gene dosage (homozygous with 2DQB1*02 alleles of the DQ2.5 haplotype), gene configuration (DQ2.2 with 2 DQB1*02 or DQ2.2 with DQ2.5 in *trans* configuration) could give additional information on CD risk in this population.

### 3.3. HLA-DQA1-DQB1-DRB1 haplotypes in populations and in CD 

Haplotype frequencies in Moroccans and in other populations are reported in Figure 2.

The high frequency of the DQ2 haplotype [6] was confirmed in the Maghreb area. Both Moroccans from the North (Metalsa) and Souss [15] showed the highest frequency of the HLA-DRB1*03-DQA1*05-DQB1*02 haplotype, as compared with other populations (Figure 2A) and the highest frequency of the DQ2 (2.5 + 2.2) haplotype (Figure 2A: Moroccans 30.2%–37.4% vs. 10.7%–12.9% of Sub-Saharans, p_c_ < 0.001; other populations: 15%–25%).

Table 2 and Table 3 show HLA-DQA1-DQB1-DRB1 haplotype frequencies in CD.

The significant association of HLA-DQ2 with CD was confirmed in Moroccan patients (Table 2). DQ2 and DQ8 haplotypes show a constant frequency in CD patients among populations (DQ2: range 58%–70%; DQ8: range 5%–8% from Morocco to Poland (Table 3) [17].

## 4. Discussion

### 4.1. CD Prevalence and Geographic Area

Prevalence of CD in North Africa is increasing; in the past it was underestimated, but in the last 10–15 years it reached around 1%, as observed in Western countries [1], that is considered the worldwide prevalence of CD. However, with the spread of diagnostic and screening strategies, increased awareness of the disease, and evaluation of subjects with mild or atypical symptoms, CD prevalence could be even greater in North Africa [18]. In this study, HLA frequencies of both North Africans and Sub-Saharans, where populations exhibit low frequencies of HLA-DQ predisposing genotypes [19,20], were included in the comparisons to evaluate the relevance of HLA genetic risk on CD development in the Maghreb area.

### 4.2. Genetic Test and DQ2 Homozygosity

From literature data, it seems that the relative risk for celiac disease associated with genotypes could be different among different geographic areas (i.e., North and South Europe) [8,21].

This study evidenced that distribution of HLA class II allelic groups and haplotypes was similar among the various Moroccan populations; very small differences in frequencies of haplotypes involved in CD susceptibility have been found between the Moroccan healthy controls employed in this study and the mixed Moroccan population considered in a recent study [5] (some variability existed only for HLA class I, HLA-A, -B, and haplotypes/associations in Moroccans), confirming previous findings [2]. A high frequency of DQ2 and DQ8 in the Moroccan population was found, 54.2%, as compared with the 20%–40% of other populations.

### 4.3. HLA-DQ2.5 Homozygosity

It is known that HLA-DQ2.5 homozygous individuals have a higher risk of CD development compared to HLA-DQ2.5/x heterozygous individuals, due to a higher expression of HLA-DQ2.5 on antigen-presenting cells (APC) and a more efficient gluten presentation [22]; recently, an effect of DQB1*02 homozygosity on CD severity has been evidenced [23]. In addition, different CD-associated DQ variants (DQ2.5, DQ2.2, and DQ7.5) confer a different risk for CD, due to the different gluten peptide sets that were selected for presentation to CD4+ T cells, as the DQ variants have different peptide-binding motifs [24].

A comparison of HLA class II genotype and haplotype frequencies was performed, both in the control populations and in CD. The HLA class II high risk genotypes (Group 1 genotypes: DQ2 with DQB1*02/02, DR3-DQ2, or DR3/DR7-DQ2) were confirmed in Moroccan patients with CD. A high frequency of DQ2 homozygous (45.2%) was evidenced in Moroccan patients. A lower contribution of DQ2.5 heterozygous, as compared with other populations, was also found.

DQ2 homozygosity increased the risk for CD (from 2% for DQ8 heterozygous to 28% for DQ2.5/DQ2.2 + 2.5 homozygous in patients at risk for CD from USA) [25].

This greater risk could be a consequence of a different level of expression of DQ2 heterodimers, as it has been observed that the anti-gluten CD4+ T cell immune response depends on the antigen dose. Anyway, a recent study concluded that neither gene dosage nor the preferential expression of CD-associated alleles (DQA1*05 and DQB1*02), as compared with non-CD-associated alleles, are able to fully explain the different disease risk, and the contribution of other genetic factors should be taken into account [26].

In addition, the identification of HLA-DQ2 homozygosity could help to predict the clinical evolution [21], as DQB1*02 homozygosity has been variably associated with some clinical forms (severe [23], refractory, and enteropathy-associated T cell lymphoma [27,28]). Results of the present study evidenced that assessing the homozygous status could be of particular interest in this area.

A possible high risk (1:10) of the DR7/DQ2 combination (α5β2 *trans* configuration, Group 2) was present in Moroccans with CD. This group should be evaluated in a larger sample for confirmation; however, results indicate the need to further investigate the expression of the different HLA-DQ heterodimers.

### 4.4. DQ8

Different molecular processes regulate deamidation and selection of gluten peptides associated with DQ2 and/or DQ8 molecules [29], thus eliciting different T cell responses. These mechanisms are at the basis of different gluten reactivity and CD risk in HLA-DQ2 or DQ8 positive patients. Comparison of DQ2 and DQ8 genotype distribution in CD did not evidence differences among populations (see results and Figure 1, respectively). As for DQ8, CD risk was lower in Moroccans and Libyans than in other populations, because of the high frequency of DQ8 in the control populations. The risk for DQ8+ DQ2.5− subjects (1:190) in Moroccans was four times lower than DQ2.5+ DQ8− (1:50) and comparable to that of DQ2.5− DQ2.2+ (1:200) or α5+ positive patients (1:170) (Table 1).

As mild/potential CD could be associated with a lower frequency of HLA-DQ2 homozygosity and increased frequency of DQB1*0302 (DQ8+) [23,30], results confirm that this study mostly included cases with overt CD, while borderline diagnoses (i.e., mild CD) have had marginal or no impact.

### 4.5. Environmental Factors and CD-Associated Genotypes

Geographical differences in CD prevalence could be due to combinations of both population-related genetic and environmental factors. Many questions are still open regarding the role and interactions between genetic and environmental factors on CD onset. Environmental factors are mainly associated with gluten introduction (timing, quantity, breastfeeding, etc.), but novel hypotheses are rising on a positive selection of CD-associated genotypes due to protection from gluten-associated negative effects: recently, a protective effect of HLA-DQ2 from dental caries onset has been suggested [31], although not demonstrated. These novel aspects (if and how DQ2 could confer resistance to other diseases associated with wheat consumption) need to be elucidated with further targeted studies.

Associations have been found between intestinal microbiota, immune/cytokine response, and HLA-DQ susceptibility haplotypes, which would affect intestinal bacterial selection [32,33].

## 5. Conclusions

We assessed CD-associated HLA-DQ-DR frequencies in Moroccan patients and controls to evaluate possible effects of different genetic substrates. Even if many theories and environmental risk factors are subject to ongoing verification—like gluten introduction in populations, timing of gluten introduction in infants, breastfeeding, composition of microbiota, metabolic profiles, vaccination schedule, infections, use of antibiotics, etc.—the high-risk HLA genotypes (DR3-DQ2 haplotype with a gene-dose effect) at present remain the most important factor affecting CD onset [34,35]. The classical HLA-DQ associations were confirmed in Moroccans with CD, with a marked tendency to share some characteristics with the populations at increased prevalence of CD. In particular, the high frequency of homozygous DQ2 and the lower risk conferred by DQ8, compared with other populations, were noteworthy.

This study confirms the need to learn more about associations with additional (i.e., non-classical) HLA and non-HLA genetic factors.

## Figures and Tables

**Table 1:** Celiac disease (CD)-associated human leukocyte antigen (HLA) class II genotypes, haplotypes, and corresponding disease risk according to Margaritte-Jeannin [8] and Megiorni [9] classification, in Moroccans and comparison with Italian [9] and Spanish [14] genetic risk for CD. The gray background shows the risk gradient associated with genotypes/haplotypes: dark gray = higher risk; light gray = lower risk; white = low or no risk.

	HLA Class II Haplotypes ^1^				DQB1*02 (α^5^β*^2^* *cis/trans* Heterodimer)	Risk Group ^2^	Risk Group ^3^	Morocco CD		Morocco Controls		CD Risk ^4^			
DQ Heterodimer	DQA1	DQB1	DRB1	DR-DQ				*n*	%	*n*	%	Morocco		Italy [9]	Spain [14]
**2DQ2 ** (*DQ2.5*) **CM^5^: 45.2%** **Risk 1:14**	**05**	**02**	**03**	**DR3-DQ2**	**Homozygous**	DQ2, DQB1*02/02	**1**	**22**	**19.1**	**3**	**3.1**	**1:14**		**1:10**	**1:12**
	**05**	**02**	**03**												
	**05**	**02**	**03**	**DR3-DQ2**	**Homozygous**			**30**	**26.1**	**3**	**3.1**				
	**0201**	**02**	**07**	**DR7-DQ2**											
**DQ2 ** (*DQ2.5*) **CM^5^: 28.8%** **Risk 1:80**	**0201**	**02**	**07**	**DR7-DQ2**	**One (α^5^β^2^*trans*)**	DQ2, DQB1*02/X	**2**	**11**	**9.6**	**1**	**1.0**	**1:10**		**1:35**	**1:64**
	**05**	**0301/04**	**11/12**	**DR11/12-DQ7**											
	**05**	**02**	**03**	**DR3-DQ2**	**Two (α^5^β^2^*cis* + *trans*)**		**3**	**8**	**7.0**	**4**	**4.2**	**1:60**	**1:83**		
	**05**	**0301/04**	**11/12**	**DR11/12-DQ7**											
	**05**	**02**	**03**	**DR3-DQ2**	**One (α^5^β^2^*cis*)**			**14**	**12.2**	**18**	**18.8**	**1:150**			
	**other**	**other**	**other**	**other**											
**DQ2 + DQ8 ** (*DQ2.5 + DQ8*) **CM^5^: 4.3%** **Risk 1:170**	**05**	**02**	**03**	**DR3-DQ2**	**One (α^5^β ^2^*cis*)**	**DQ2 and DQ8**	**3**	**5**	**4.3**	**7**	**7.3**	**1:170**		**1:7**	**1:22**
	**03**	**0302**	**04**	**DR4-DQ8**											
**DQ8 ** **CM^5^: 8.7%** **Risk 1:190**	**0201**	**02**	**07**	**DR7-DQ2.2**		DQ8, DQB1*02+	**4**	**2**	**1.7**	**2**	**2.1**	**1:120**		**1:24**	**1:22**
	**03**	**0302**	**04**	**DR4-DQ8**											
	**03**	**0302**	**04**	**DR4-DQ8**		DQ8, DQB1*02−	**4/5**	**8**	**7.0**	**14**	**14.6**	**1:200**		**1:89**	**1:265**
	**other**	**other**	**other**												
**DQ2.2 ** (*half DQ2*) **CM^5^: 7.8%** **Risk 1:200**	**0201**	**02**	**07**	**DR7-DQ2.2**	Homozygous	β2, DQB1*02/02	**4**	**2**	**1.7**	**1**	**1.0**	**1:59**		**1:26**	**1:1063**
	**0201**	**02**	**07**												
	**0201**	**02**	**07**	**DR7-DQ2.2**		β2, DQB1*02/X	**5**	**7**	**6.1**	**12**	**12.5**	**1:205**		**1:210**	
	**other**	**other**	**other**												
**Other^4^** **CM^5^: 5.2%** **Risk > 1:200**	**05**	**0301/04**	**11/12**	**DR11/12-DQ7**		α5 (DQA1*05)	**5**	**4**	**3.5**	**7**	**7.3**	**1:209**		**1:1842**	**-**
	**other**	**other**	**other**												
	**05**	**other**	**other**					**2**	**1.7**	**2**	**2.1**	**1:124**			
	**other**	**other**	**other**												
	**other**	**other**	**other**					**0**	**0**	**22**	**22.9**	**-**		**1:2518**	**-**
	**other**	**other**	**other**												
**Total**								**115**	**100**	**96**	**100**				

^1^ other = non-CD-associated alleles; ^2^ according to [9]: DQ2 = DQA1*05, DQB1*02; β2 chain: presence of only one chain (β2) of the DQ2 heterodimer; ^3^ according to [8]; ^4^ risk considering a prevalence of 1:100; ^5^ CM: Moroccans with CD.

**Table 2:** Distribution of the *HLA-DQA1-DQB1-DRB1* haplotypes in CD in Moroccans.

		Controls (2*n* = 192)	Celiac Disease (2*n* = 230)	
*DQA1-DQB1-DRB1*		Frequency (*n*)	Frequency (*n*)	*p*
**DQ2**	*05-*02-*03 (DQ2.5)	0.20 (38)	0.44 (101)	*p*_c_ < 0.0000
				OR: 3.17
				CI: 2.04–4.93
	*02-*02-*07 (DQ2.2)	0.10 (20)	0.24 (54)	*p*_c_ = 0.003
				O.R. = 2.64
				CI: 1.52–4.59
**DQ8**	*x-*03:02-*04	0.14 (26)	0.06 (15)	ns
*05-*03:01-*11/12		0.06 (12)	0.10 (23)	ns
*05-*x-*x, *x-*x-*x ^1^		0.50 (96)	0.16 (37)	*p*_c_ < 0.0000
				OR: 0.19
				CI: 0.12–0.30

^1^ haplotypes not associated with CD risk. OR: odds ratio; CI: confidence interval; ns: not significant.

**Table 3:** Frequency distribution of *HLA-DQA1-DQB1-DRB1* haplotypes in celiac disease (CD) in Moroccans and in European populations.

	*DQA1-DQB1-DRB1*	Morocco [present study]	Spain [17]	Greece [12]	Italy [17]	UK [17]	The Netherlands [17]	Poland [17]
**DQ2**	*05-*02-*03	0.44	0.45	0.40	0.32	0.52	0.54	0.39
	**(DQ2.5)**							
	*02-*02-*07	0.24	0.23	0.20	0.28	0.16	0.13	0.19
	**(DQ2.2)**							
**DQ8**	*x-*03:02-*04	0.06	0.06	0.08	0.05	0.06	0.06	0.07
*05-*03:01-*11/12		0.10	0.09	0.16	0.18	0.03	0.05	0.10
*05-*x-*x, *x-*x-*x ^1^		0.16	0.17	0.15	0.17	0.23	0.22	0.25

^1^ haplotypes not associated with CD risk.

## References

[1] Greco L., Timpone L., Abkari A., Abu-Zekry M., Attard T., Bouguerrà F., Cullufi P., Kansu A., Micetic-Turk D., Mišak Z. (2011). Burden of celiac disease in the Mediterranean area. World J. Gastroenterol..

[2] Piancatelli D., Canossi A., Aureli A., Oumhani K., del Beato T., Di Rocco M., Liberatore G., Tessitore A., Witter K., El Aouad R. (2004). Human leukocyte antigen-A, -B, and -Cw polymorphism in a Berber population from North Morocco using sequence-based typing. Tissue Antigens.

[3] Nunes J.M., Buhler S., Roessli D., Sanchez-Mazas A., The HLA-net 2013 Collaboration (2014). The HLA-net GENE[RATE] pipeline for effective HLA data analysis and its application to 145 population samples from Europe and neighbouring areas. Tissue Antigens.

[4] Li X., Ghandri N., Piancatelli D., Adams S., Chen D., Robbins F., Wang E., Monaco A., Selleri S., Bouaouina N. (2007). Associations between HLA Class I alleles and the prevalence of nasopharyngeal carcinoma (NPC) among Tunisians. J. Transl. Med..

[5] Brick C., Atouf O., Bouayad A., Essakalli M. (2015). Moroccan study of HLA (-A, -B, -C, -DR, -DQ) polymorphism in 647 unrelated controls. Updating data. Mol. Cell. Probes.

[6] Cataldo F., Montalto G. (2007). Celiac disease in the developing countries: A new and challenging public health problem. World J. Gastroenterol..

[7] Megiorni F., Mora B., Bonamico M., Nenna R., Di Pierro M., Catassi C., Drago S., Mazzilli M.C. (2008). A rapid and sensitive method to detect specific human lymphocyte antigen (HLA) class II alleles associated with celiac disease. Clin. Chem. Lab. Med..

[8] Margaritte-Jeannin P., Babron M.C., Bourgey M., Louka A.S., Clot F., Percopo S., Coto I., Hugot J.P., Ascher H., Sollid L.M. (2004). HLA-DQ relative risks for coeliac disease in European populations: A study of the European Genetics Cluster on Coeliac Disease. Tissue Antigens.

[9] Megiorni F., Mora B., Bonamico M., Barbato M., Nenna R., Maiella G., Lulli P., Mazzilli M.C. (2009). HLA-DQ and risk gradient for celiac disease. Hum. Immunol..

[10] Excoffier L., Lischer H.E.L. (2010). Arlequin suite ver 3.5: A new series of programs to perform population genetics analyses under Linux and Windows. Mol. Ecol. Resour..

[11] Alarida K., Harown J., Di Pierro M.R., Drago S., Catassi C. (2010). HLA-DQ2 and -DQ8 genotypes in celiac and healthy Libyan children. Dig. Liver Dis..

[12] Krini M., Chouliaras G., Kanariou M., Varela I., Spanou K., Panayiotou J., Roma E., Constantinidou N. (2012). HLA class II high-resolution genotyping in Greek children with celiac disease and impact on disease susceptibility. Pediatr. Res..

[13] Hernandez-Charro B., Donat E., Miner I., Aranburu E., Sánchez-Valverde F., Ramos-Arroyo M.A. (2008). Modifying effect of HLA haplotypes located trans to DQB1*02-DRB1*03 in celiac patients of Southern Europe. Tissue Antigens.

[14] Ruiz-Ortiz E., Montraveta M., Cabré E., Herrero-Mata M.J., Pujol-Borrell R., Palou E., Faner R. (2014). HLA-DQ2/DQ8 and HLA-DQB1*02 homozygosity typing by real-time polymerase chain reaction for the assessment of celiac disease genetic risk: Evaluation of a Spanish celiac population. Tissue Antigens.

[15] Gonzalez-Galarza F.F., Takeshita L.Y., Santos E.J., Kempson F., Maia M.H., Silva A.L., Silva A.L., Ghattaoraya G.S., Alfirevic A., Jones A.R. (2015). Allele frequency net 2015 update: New features for HLA epitopes, KIR and disease and HLA adverse drug reaction associations. Nucleic Acid Res..

[16] Bourgey M., Calcagno G., Tinto N, Gennarelli D., Margaritte-Jeannin P., Greco L., Limongelli M.G., Oscar Esposito O., Marano C., Troncone R. (2007). HLA related genetic risk for coeliac disease. Gut.

[17] Gutierrez-Achury J., Zhernakova A., Pulit S.L., Trynka G., Hunt K.A., Romanos J., Raychaudhuri S., van Heel D.A., Wijmenga C., de Bakker P.I. (2015). Fine mapping in the MHC region accounts for 18% additional genetic risk for celiac disease. Nat. Genet..

[18] Barada K., Bitar A., Mokadem M.A., Hashash J.G., Green P. (2010). Celiac disease in Middle Eastern and North African countries: A new burden?. World J. Gastroenterol..

[19] Cataldo F., Lio D., Simpore J., Musumeci S. (2002). Consumpion of wheat foodstuffs not a risk for celiac disease occurrence in Burkina Faso. J. Pediatr. Gastroenterol. Nutr..

[20] Cataldo F., Pitarresi N., Accomando S., Greco L., SIGENP, GLNBI Working Group on Coeliac Disease (2004). Epidemiological and clinical features in immigrant children with coeliac disease: An Italian multicentre study. Dig. Liver Dis..

[21] Murray J.A., Moore S.B., van Dyke C.T., Lahr B.D., Dierkhising R.A., Zinsmeister A.R., Melton L.J., Kroning C.M., El-Yousseff M., Czaja A.J. (2007). HLA DQ gene dosage and risk and severity of celiac disease. Clin. Gastroenterol. Hepatol..

[22] Vader W., Stepniak D., Kooy Y., Mearin L., Thompson A., van Rood J.J., Spaenij L., Koning F. (2003). The HLA-DQ2 gene dose effect in celiac disease is directly related to the magnitude and breadth of gluten-specific T cell responses. Proc. Natl. Acad. Sci. USA.

[23] Biagi F., Vattiato C., Marchese A., Trotta L., Badulli C., de Silvestri A., Martinetti M., Corazza G.R. (2012). Influence of HLA-DQ2 and DQ8 on severity in celiac disease. J. Clin. Gastroenterol..

[24] Bergseng E., Dørum S., Arntzen M.Ø., Nielsen M., Nygård S., Buus S., de Souza G.A., Sollid L.M. (2015). Different binding motifs of the celiac disease-associated HLA molecules DQ2.5, DQ2.2, and DQ7.5 revealed by relative quantitative proteomics of endogenous peptide repertoires. Immunogenetics.

[25] Pietzak M.M., Schofield T.C., McGinniss M.J., Nakamura R.M. (2009). Stratifying risk for celiac disease in a large at-risk United States population by using HLA alleles. Clin. Gastroenterol. Hepatol..

[26] Pisapia L., Camarca A., Picascia S., Bassi V., Barba P., del Pozzo G., Gianfrani C. (2016). HLA-DQ2.5 genes associated with celiac disease risk are preferentially expressed with respect to non-predisposing HLA genes: Implication for anti-gluten T cell response. J. Autoimmun..

[27] Karinen H., Kärkkäinen P., Pihlajamäki J., Janatuinen E., Heikkinen M., Julkunen R., Kosma V.M., Naukkarinen A., Laakso M. (2006). Gene dose effect of the DQB1*0201 allele contributes to severity of coeliac disease. Scand. J. Gastroenterol..

[28] Al-Toma A., Goerres M.S., Meijer J.W., Peña A.S., Crusius J.B., Mulder C.J. (2006). Human leukocyte antigen-DQ2 homozygosity and the development of refractory celiac disease and enteropathy-associated T-cell lymphoma. Clin. Gastroenterol. Hepatol..

[29] Henderson K.N., Tye-Din J.A., Reid H.H., Chen Z., Borg N.A., Beissbarth T., Tatham A., Mannering S.I., Purcell A.W., Dudek N.L. (2007). A structural and immunological basis for the role of human leukocyte antigen DQ8 in celiac disease. Immunity.

[30] Schirru E., Jores R.D., Cicotto L., Frau F., de Virgiliis S., Rossino R., Macis M.D., Lampis R., Congia M. (2011). High frequency of low-risk human leukocyte antigen class II genotypes in latent celiac disease. Hum. Immunol..

[31] Valarini N., Maciel S.M., Moura S.K., Poli-Frederico R.C. (2012). Association of Dental Caries with HLA Class II Allele in Brazilian Adolescents. Caries Res..

[32] Cicerone C., Nenna R., Pontone S. (2015). Th17, intestinal microbiota and the abnormal immune response in the pathogenesis of celiac disease. Gastroenterol. Hepatol. Bed Bench.

[33] Olivares M., Neef A., Castillejo G., Palma G.D., Varea V., Capilla A., Palau F., Nova E., Marcos A., Polanco I. (2015). The HLA-DQ2 genotype selects for early intestinal microbiota composition in infants at high risk of developing coeliac disease. Gut.

[34] Lionetti E., Castellaneta S., Francavilla R., Pulvirenti A., Tonutti E., Amarri S., Barbato M., Barbera C., Barera G., Bellantoni A. (2014). Introduction of gluten; HLA status; and the risk of celiac disease in children. N. Engl. J. Med..

[35] Liu E., Lee H.S., Aronsson C.A., Hagopian W.A., Koletzko S., Rewers M.J., Eisenbarth G.S., Bingley P.J., Bonifacio E., Simell V. (2014). Risk of Pediatric Celiac Disease According to HLA Haplotype and Country. N. Engl. J. Med..

